# Mibefradil and Flunarizine, Two T-Type Calcium Channel Inhibitors, Protect Mice against Lipopolysaccharide-Induced Acute Lung Injury

**DOI:** 10.1155/2020/3691701

**Published:** 2020-11-10

**Authors:** Limei Wan, Weibin Wu, Shunjun Jiang, Shanhe Wan, Dongmei Meng, Zhipeng Wang, Jiajie Zhang, Li Wei, Pengjiu Yu

**Affiliations:** ^1^The First Affiliated Hospital of Guangdong Pharmaceutical University, Guangzhou 510080, China; ^2^Department of Basic Medicine, Zhaoqing Medical College, Zhaoqing 526020, China; ^3^Department of Pharmacy, The First Affiliated Hospital of Guangzhou Medical University, Guangzhou 510120, China; ^4^Guangdong Provincial Key Laboratory of New Drug Screening, School of Pharmaceutical Science, Southern Medical University, Guangzhou 510515, China

## Abstract

Recent studies have illuminated that blocking Ca^2+^ influx into effector cells is an attractive therapeutic strategy for lung injury. We hypothesize that T-type calcium channel may be a potential therapeutic target for acute lung injury (ALI). In this study, the pharmacological activity of mibefradil (a classical T-type calcium channel inhibitor) was assessed in a mouse model of lipopolysaccharide- (LPS-) induced ALI. In LPS challenged mice, mibefradil (20 and 40 mg/kg) dramatically decreased the total cell number, as well as the productions of TNF-*α* and IL-6 in bronchoalveolar lavage fluid (BALF). Mibefradil also suppressed total protein concentration in BALF, attenuated Evans blue extravasation, MPO activity, and NF-*κ*B activation in lung tissue. Furthermore, flunarizine, a widely prescripted antimigraine agent with potent inhibition on T-type channel, was also found to protect mice against lung injury. These data demonstrated that T-type calcium channel inhibitors may be beneficial for treating acute lung injury. The important role of T-type calcium channel in the acute lung injury is encouraged to be further investigated.

## 1. Introduction

Acute lung injury (ALI) is a life-endangering syndrome featured by serious lung inflammation and noncardiogenic pulmonary edema; acute respiratory distress syndrome (ARDS) presents the most severe form of ALI [1]. Severe bacterial infection is one of the most common contributors of ALI/ARDS [2]. Although various protective strategies including extracorporeal membrane oxygenation (ECMO), prone position ventilation (PPV), and continuous high-volume hemofiltration (CHVH) have been wildly used, the mortality of ALI/ARDS is still unacceptable [3, 4]. Thus, novel effective medicines and a more meaningful intelligence of the underlying pathogenic mechanisms are urgently required.

Recent studies have illuminated the crucial role of calcium in the occurrence and development of ALI [5–7]. An increase in intracellular Ca^2+^ gives rise to transformations in endothelial cell morphology and the expanding of adherent junctions, leading to increasing of endothelial permeability [8, 9]. The Ca^2+^ oscillations are also involved in controlling neutrophil activation and endothelial cellular inflammatory responses, including regulation of gene expression and cell death, which are mainly modulated by NF-*κ*B [10–12]. Therefore, blocking calcium influx into effector cells is an attractive therapeutic strategy for lung injury, since it yields remission in both increases of endothelial permeability and neutrophilic inflammation.

T-type calcium channels are low-voltage-activated channels, which contain three different subunits: *α*_1_G, *α*_1_H, and *α*_1_I, also known as Ca_v_3.1, Ca_v_3.2, and Ca_v_3.3, respectively [13]. The physical roles of T-type channels have been reported in different tissues, such as smooth muscle contraction [14], fertilization [15], pain neurotransmission [16], pacing of the heart [17], or adrenal steroid biosynthesis [18]. The effects of T-type calcium channels in pulmonary microvascular endothelium have been also investigated [19, 20]. Wu et al. demonstrated that Ca_v_3.1 channel is expressed in lung microvascular endothelial cells, while lung macrovascular endothelial cells do not express it [21]. Importantly, Ca_v_3.1 channel has been reported to regulate the expressions of P-selectin and vWF in pulmonary microvascular endothelial cells [22, 23].

In this study, we hypothesize that T-type calcium channel is a potential target for treating ALI. A lipopolysaccharide- (LPS-) induced ALI mice model was used because of its reproducibility and handleability characteristics. LPS exposure causes a rapid influx of neutrophils, overwhelming release of inflammatory cytokines, and severe protein leakage in the lung, which admirably mimic the pathophysiologic alterations observed in ALI/ARDS patients [24]. Mibefradil is an acknowledged T-type calcium channel inhibitor that was first launched on the market as antihypertensive and antianginal agent [25]. We explored the potentially protective role of mibefradil on LPS-induced lung injury model. In addition, the protective effect of flunarizine, an antimigraine agent with potent inhibition of T-type calcium channel, was further evaluated.

## 2. Materials and Methods

Mibefradil dihydrochloride (Purity: 98.49% by LC-MS) was purchased from MedChem Express (Shanghai, China). Flunarizine hydrochloride and LPS (Escherichia coli 055:B5) were purchased from Sigma-Aldrich (St, Louis, MO, USA). ELISA kits for examination of mouse TNF-*α* and IL-6 were purchased from Dakewe Biotech Co. Ltd (Beijing, China). Antibodies for phosphorylated p65 and *β*-actin were purchased from Cell Signaling Technology (Danvers, Massachusetts, USA).

### 2.1. Animals and Procedures

All animal care and experimental procedures were abided by the National Institutes of Health Guidelines for the Care and Use of Laboratory Animals and were approved by The Medical Ethics Committee of The First Affiliated Hospital of Guangzhou Medical University.

Male BALB/c mice (6-8 weeks old; 18-22 g) were obtained from Experimental Animal Center of Guangdong province (Foshan city, China) and were housed in standardized conditions in animal facilities at 20 ± 2°C room temperature, 40 ± 5% relative humidity with a 12 h light/dark cycle. LPS-induced ALI was processed as described in our previous study [26]. Mice were placed in a plexiglass chamber (20 × 30 × 40 cm) throughout the LPS exposure (30 min). LPS solution (0.5 mg/mL) was aerosolized through an ultrasonic nebulizer (NB-150U, Omron Co., Kyoto, Japan).

Mibefradil was dissolved in saline. In a set of experiments to investigate the effects of flunarizine, the solvent is distilled water. Drugs were freshly prepared and intraperitoneally injected 30 min before or after LPS exposure. The dosages of mibefradil (20 and 40 mg/kg) [27, 28] and flunarizine (30 mg/kg) [29] were according to the previous studies. Mice were sacrificed 6 h after end of LPS exposure.

Bronchoalveolar lavage fluid (BALF) collection for total cell count, as well as measurements of total protein concentration and cytokines level, BALF collection was performed as our previously described [26]. Briefly, after tracheostomy was processed, a cannula was placed into the trachea and tightened with surgical silks; the lungs were lavaged 3 times with cold PBS (0.5 mL for each time). A part of BALF (0.1 mL) was used for the total cell counting by using a hemocytometer; the rest was centrifuged at 500 g for 10 min at 4°C. Total protein concentration and the levels of TNF-*α* and IL-6 in the supernatant were measured.

### 2.2. Evans Blue Assay

To further test the protein leakage, Evans blue dye- (EBD-) albumin conjugate (0.5% EBD/4% BSA solution in saline) was injected through the tail vein (30 mg/kg) 30 min before sacrifice. Mice were killed by an overdose of pentobarbitone (200 mg/kg, i.p); then, the EBD in the systemic circulation system was rinsed with saline. After that, lungs were excised then placed in 2 mL formamide to extract EBD (72 h, 42°C). Optical density was examined at 620 nm, and the EBD concentration was calculated with expression as *μ*g/g of tissue.

### 2.3. Histological Evaluation and MPO Activity Measurement

Left lobe was fixed with 10% formalin for 48 h and then embedded in paraffin. Sections with 5 *μ*m thick were stained with hematoxylin and eosin. Lung injury score was performed as described by previous study [30]: (1) alveolar congestion, (2) hemorrhage, (3) infiltration or aggregation of neutrophils in airspace or vessel wall, and (4) thickness of the alveolar wall. For each subject, a five-point scale was applied: 0, minimal (little) damage; 1+, mild damage; 2+, moderate damage; 3+, severe damage; and 4+, maximal damage. Points were added up and are expressed as median ± rangeofinjuryscore.

The rest of lung lobes were homogenized in PBS; MPO activity in the homogenate was measured according to the manufacturer's instruction (Nanjing JianCheng Bioengineering Institute, Nanjing, China) and was expressed as units per gram of protein.

### 2.4. Western Blot

The total protein was extracted from lung tissues, and protein concentration was measured by the BCA method. Protein samples were solubilized in SDS buffer and separated on SDS-PAGE gels and then transferred to PVDF membranes. The membranes were blocked with 5% nonfat milk and then incubated with primary antibody (phosphorylated p65, p65, I*κ*B-*α* or *β*-actin) and conjugated secondary antibody in succession. ECL detection kit (Millipore, Billerica, USA) was used to detect protein bands, and the protein signals were quantified.

### 2.5. Statistical Analysis

The SPSS 13.0 software was used for data analysis. All values are expressed as means ± standarderrorofthemean (SEM). Data were analyzed by using one-way analysis of variance followed by LSD test. Two-tailed *p* values < 0.05 were considered statistically significant.

## 3. Results

### 3.1. Mibefradil Decreased Cell Counts and Inflammatory Cytokines Level in BALF of LPS Challenged Mice

**Figure 1:**
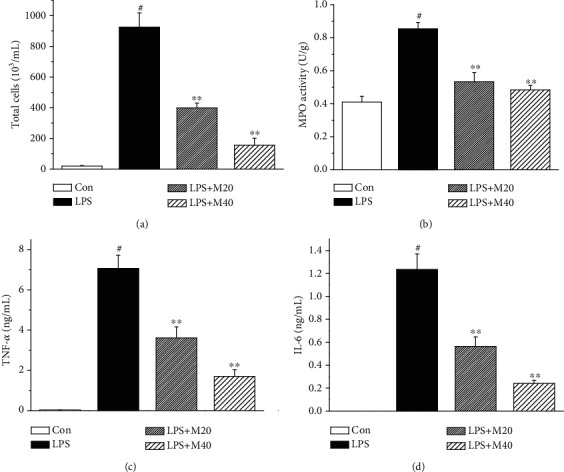
Mibefradil decreased cell counts and inflammatory cytokines level in BALF of LPS-induced ALI mice. Mibefradil (20 and 40 mg/kg) was administrated 30 min before LPS exposure. Mice were sacrificed 6 h after LPS exposure and bronchoalveolar lavage was processed. The total cell number (a), TNF-*α* (c), and IL-6 (d) levels in BALF were measured. (b) Six hours after LPS exposure, mice were sacrificed and the right lung tissues were homogenized with PBS for MPO assay. All values are mean ± SEM (*n* = 6). ^#^*p* < 0.05, significant compared with vehicle-treated control; ^∗^*p* < 0.05, significant compared with LPS alone; ^∗∗^*p* < 0.01, significant compared with LPS alone.

Inflammatory cell influx is a key event at the early stage of ALI. As shown in Figure 1, LPS exposure caused a remarkable cell influx into BALF. Pretreatment of 20 and 40 mg/kg mibefradil markedly suppressed LPS-induced cell influx. In addition, mibefradil also significantly lowered LPS-induced MPO activity in lung tissue, which is a key indicator of neutrophils infiltration in tissue.

We also examined the inflammatory cytokine levels in BALF. LPS exposure resulted in obviously increased levels of TNF-*α* and IL-6 in BALF, whereas these rises were dose-dependently inhibited by mibefradil.

### 3.2. Mibefradil Decreased Protein Concentration in BALF and Inhibited Evans Blue Extravasation in Lung Tissue

**Figure 2:**
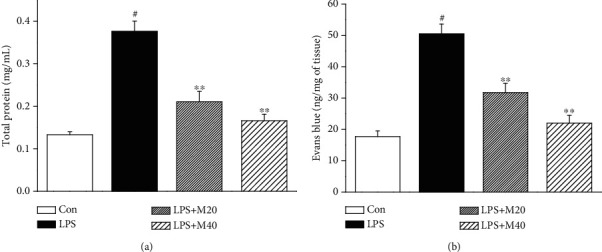
Mibefradil decreased total protein concentration in BALF and inhibited Evans blue extravasation in lung tissue. Mibefradil (20 and 40 mg/kg) was administrated 30 min before LPS exposure. Mice were sacrificed 6 h after LPS challenge and bronchoalveolar lavage was processed. (a) The concentration of total protein in BALF was measured. (b) Evans blue dye (30 mL/kg, *i*/*v*) was injected 0.5 h before sacrifice. Evans blue accumulation in the lung tissue was examined to test pulmonary vascular permeability. All values are mean ± SEM (*n* = 6). ^#^*p* < 0.05, significant compared with vehicle-treated control; ^∗^*p* < 0.05, significant compared with LPS alone; ^∗∗^*p* < 0.01, significant compared with LPS alone.

Vascular leakage is a crucial event of lung injury; therefore, we measured the total protein level in BALF. As shown in Figure 2, LPS exposure caused a dramatic elevation of protein concentration in BALF, from 0.133 ± 0.007 to 0.376 ± 0.024 mg/mL. Pretreatment with 20 and 40 mg/kg mibefradil significantly inhibited total protein level in BALF of LPS challenged mice. In parallel with the total protein levels, pretreatment with mibefradil also suppressed LPS-induced increase in Evans blue extravasation.

### 3.3. Mibefradil Attenuated LPS-Induced Pathological Alterations in Lung Tissues

**Figure 3:**
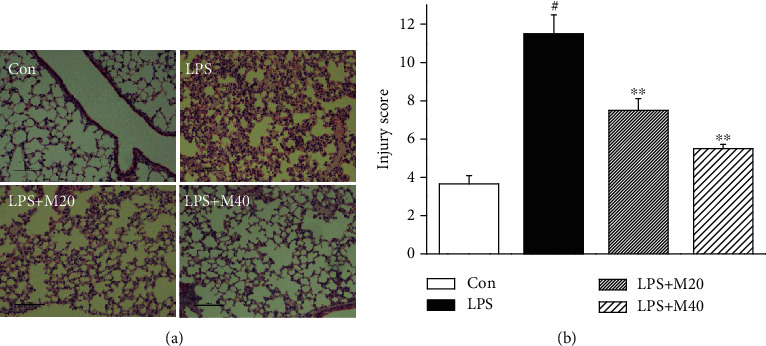
Mibefradil attenuated histological changes in lung tissues of LPS-induced ALI mice. Mibefradil (20 and 40 mg/kg) was treated 0.5 h before LPS challenge. Mice were sacrificed 6 h after LPS exposure. The left lung was fixed, embedded in paraffin, and cut into 5 *μ*m slices. Histological assay was conducted by light microscopy after H&E staining (a), and lung injury was scored (b). All values are mean ± SEM (*n* = 6). ^#^*p* < 0.05, significant compared with vehicle-treated control; ^∗^*p* < 0.05, significant compared with LPS alone; ^∗∗^*p* < 0.01, significant compared with LPS alone.

The pulmonary histopathology was evaluated by HE staining and lung injury score system. Compared with control group, lung sections in mice treated with LPS showed notable neutrophils infiltration, alveolar hemorrhage, and interalveolar septal thickening. Treatment with mibefradil improved pulmonary histological changes in LPS challenged mice (Figure 3).

### 3.4. Mibefradil Inhibited LPS-Induced NF-*κ*B Activation in Lung Tissues

**Figure 4:**
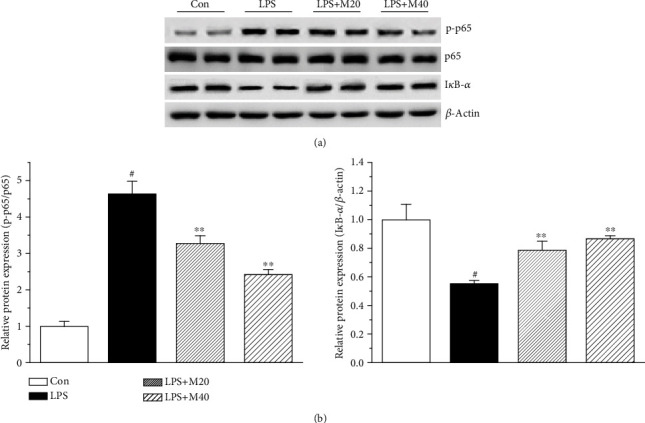
Mibefradil inhibited p65 phosphorylation and I*κ*B-*α* degradation in lung tissues of LPS-induced ALI mice. Mibefradil (40 mg/kg) was administrated 30 min before LPS exposure. Mice were sacrificed 6 h after LPS exposure, and the whole protein extraction from lung tissues was processed. The phosphorylated p65 and I*κ*B-*α* expressions were measured by Western blotting, and the protein signals were quantified. All values are mean ± SEM (*n* = 4). ^#^*p* < 0.05, compared with vehicle-treated control; ^∗^*p* < 0.05, significant compared with LPS alone; ^∗∗^*p* < 0.01, significant compared with LPS alone.

NF-*κ*B plays a center role in the regulation of inflammation, and phosphorylation of p65 and degradative I*κ*B-*α* are key signs of NF-*κ*B activation. We measured phosphorylated p65 and I*κ*B-*α* levels in lung tissue by Western blot method. As shown in Figure 4, mibefradil inhibited phosphorylation of p65 and degradation of I*κ*B-*α*, which demonstrated that mibefradil suppressed NF-*κ*B pathway activation in lung tissues of LPS challenged mice.

### 3.5. Therapeutic Effects of Mibefradil on LPS-Induced Lung Injury

**Figure 5:**
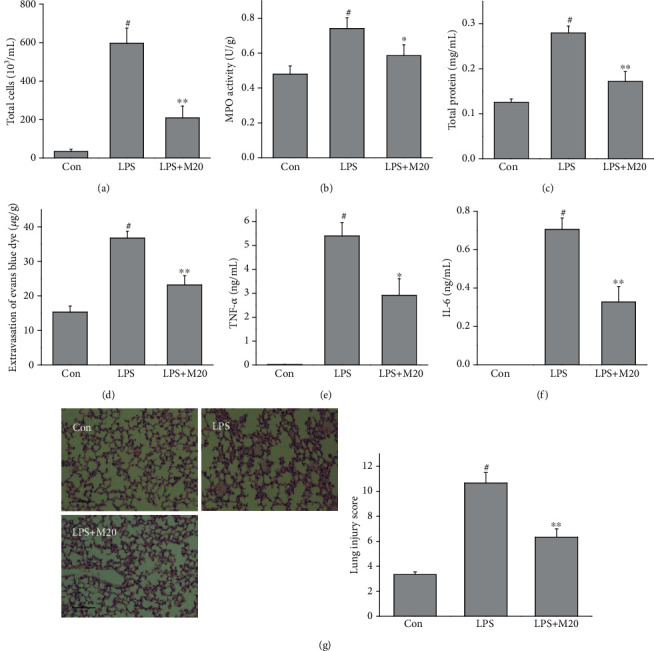
Therapeutic effects of mibefradil on LPS-induced lung injury in mice. Mibefradil (20 mg/kg) was injected 30 min after LPS exposure, and mice were sacrificed 6 h after LPS exposure. The total cell counts (a) in BALF, MPO activities (b) in lung tissue, total protein concentration (c) in BALF, extravasation of Evans blue dye (d) in lung tissue, TNF-*α* (e) and IL-6 (f) levels in BALF, and pathological changes (g) in the lung were measured. All values are mean ± SEM (*n* = 6). ^#^*p* < 0.05, compared with vehicle-treated control; ^∗^*p* < 0.05, significant compared with LPS alone; ^∗∗^*p* < 0.01, significant compared with LPS alone.

To additionally evaluate the therapeutic effects of mibefradil on LPS-induced lung injury, mice were treated with mibefradil (20 mg/kg) 30 min after LPS exposure. As Figure 5 shown, mibefradil attenuated the cell influx, protein leakage, and inflammatory cytokines release in ALI mice.

### 3.6. Flunarizine Protected Mice from LPS-Induced ALI

**Figure 6:**
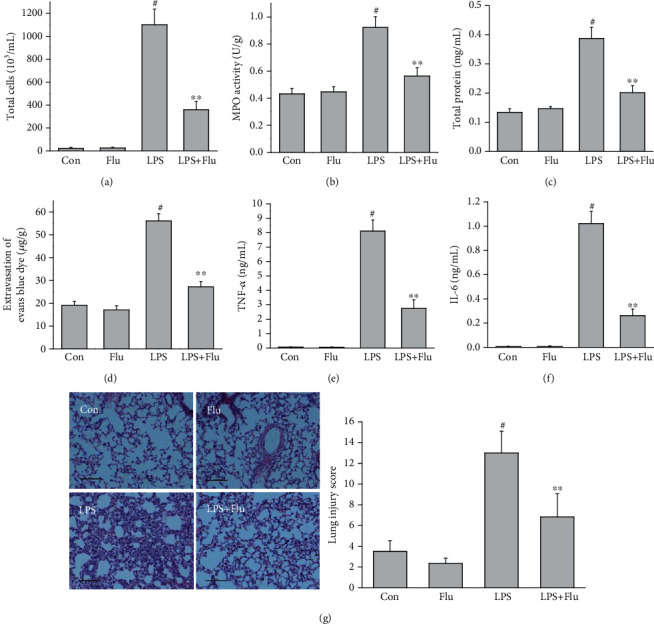
Preventive effects of flunarizine on LPS-induced ALI. Flunarizine (30 mg/kg) was treated 30 min before LPS exposure, and mice were sacrificed 6 h after LPS exposure. The total cell counts (a) in BALF, MPO activities (b) in lung tissue, total protein concentration (c) in BALF, extravasation of Evans blue dye (d) in lung tissue, TNF-*α* (e) and IL-6 (f) levels in BALF, and pathological changes (g) in the lung were measured. All values are mean ± SEM (*n* = 6). ^#^*p* < 0.05, compared with vehicle-treated control; ^∗^*p* < 0.05, significant compared with LPS alone; ^∗∗^*p* < 0.01, significant compared with LPS alone.

**Figure 7:**
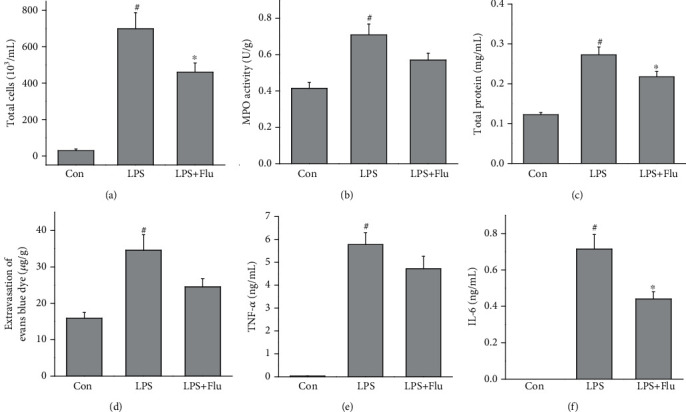
Therapeutic effects of flunarizine on LPS-induced lung injury. Flunarizine (30 mg/kg) was injected 30 min after LPS exposure, and mice were sacrificed 6 h after LPS exposure. The total cell counts (a) in BALF, MPO activities (b) in lung tissue, total protein concentration (c) in BALF, extravasation of Evans blue dye (d) in lung tissue, and TNF-*α* (e) and IL-6 (f) levels in BALF were measured. All values are mean ± SEM (*n* = 6). ^#^*p* < 0.05, compared with vehicle-treated control; ^∗^*p* < 0.05, significant compared with LPS alone; ^∗∗^*p* < 0.01, significant compared with LPS alone.

To additionally verify the protective properties of T-type calcium channel inhibitor on lung injury, we investigated the pharmacological activity of flunarizine, another proven T-type calcium channel inhibitor which has been widely prescribed for migraine prophylaxis, on LPS-induced ALI mice model. As expected, preventive treatment with 30 mg/kg flunarizine significantly suppressed the LPS-induced cell influx, protein leakage, and inflammatory cytokines release (Figure 6). Posttreatment with 30 mg/kg flunarizine also inhibited the pulmonary inflammation; however, the effectiveness was lesser than the preventive effect (Figure 7).

## 4. Discussion

In this study, we reported that mibefradil significantly decreased LPS-induced total cell number, protein concentration, and Evans blue extravasation, as well as TNF-*α* and IL-6 levels in BALF. Mibefradil also suppressed MPO activity and attenuated pathological alterations in lung tissue of LPS challenged mice. In addition, mibefradil suppressed NF-*κ*B activation, a central transcription factor regulating gene expression of various inflammatory mediators. Since inflammatory cells influx, protein leakage, and cytokine outburst are the crucial events of ALI in humans and animals [1], our results demonstrated that mibefradil protected mice against LPS-induced lung injury.

The calcium channels are now receiving more attention as novel therapeutic targets of lung injury [5, 31, 32]. In general, Ca^2+^ channels can be classified based on their activation pattern and are divided into voltage-dependent calcium channels (VDCC) and non-VDCC. VDCC channels contain L-, N-, P-, Q-, R-, and T-types, while non-VDCC channels include store-operated Ca^2+^ entry channels (SOCC), receptor-operated Ca^2+^ entry channels (ROCC), and mechanosensitive Ca^2+^ entry channels (MSCC) [33, 34]. Transient receptor potential (TRP) family is the main constituent part of non-VDCC channels; recent studies have demonstrated that blocking TRPV4 and TRPC6, two members of TRPs, resulted in significant improvement of rodent models of lung injury [7, 35–37]. Furthermore, inhibition of STIM1, a critical regulator of TRPs, also dramatically prevented mice from experimental lung injury [38]. Inspired by these initial promising results, we further investigated the potential effects of VDCC and found that pharmacological inhibition of T-type calcium exhibited marked therapeutic benefit on LPS-induced lung injury. Because lacking of selective inhibitor on Ca_v_3.1, Ca_v_3.2, or Ca_v_3.3, we did not illuminate which subunit is the primary target of lung injury. Previous studies have reported that Ca_v_3.1 forms functional T-type calcium channels in pulmonary microvascular endothelial cells, and the secretions of von Willebrand factor (vWF) as well as P-selectin were selectively regulated by Ca_v_3.1 in pulmonary capillary endothelium [19, 22, 23]. These data reminded us that inhibition of Ca_v_3.1 may be the contributor against lung injury. However, more studies of experimental lung injury models based on transgenic animal technology are needed.

Identifying new uses for existing drugs is one way to avoid the current costly and time-consuming status of drug discovery. Because existing drugs have known pharmacokinetics and safety profiles, the pharmacokinetic and toxicological experiments could probably be eliminated [39]. Although mibefradil has been withdrawn in 1998 because of severe drug interactions, there are still a few drugs with potential inhibitory effect on T-type calcium channel in the market, such as flunarizine, penfluridol, and ethosuximide [40, 41]. Flunarizine is one of the most widely prescribed medicine for migraine prevention. Additionally, flunarizine is used as a first-line medication for migraine prophylaxis in children and adolescents because of its satisfactory safety and efficacy profiles [42]. Previous studies reported that flunarizine potently inhibited T-type calcium channel in ventricular myocytes [43, 44], aorta smooth muscle cells [45], granulosa cells [46], pulmonary microvascular endothelial cells [21], and spermatogenic cells [47]. In this study, we found that preventive treatment with flunarizine significantly inhibited LPS-induced protein leakage, cell influx, and inflammatory cytokine release in BALF and improved the pathologic changes in lung tissues. These results verified the protective effect of T-type calcium channel inhibitors on lung injury. What is more, since flunarizine has acceptable safety and tolerability for long-term usage, further clinical works are warranted to explore the potential of this drug in the prevention of ALI.

## 5. Conclusion

In summary, our study demonstrated that T-type calcium channel inhibitors may be beneficial for treating lung injury. The key role of T-type calcium channel in the acute lung injury is encouraged to be further investigated.

## Data Availability

The data used to support the findings of this study are available from the corresponding author upon request.

## Supplementary Materials

Supplementary Materials Supplement figures: mibefradil (40 mg/kg) or saline was treated 30 min before aerosolized saline exposure, and mice were sacrificed 6 h after aerosol inhalation of saline. The total cell counts (a) in BALF, MPO activities (b) in lung tissue, total protein concentration (c) in BALF, extravasation of Evans blue dye (d) in lung tissue, TNF-*α* (e) and IL-6 (f) levels in BALF, pathological changes (g), and NF-*κ*B activation in the lung were measured. All values are mean ± SEM (*n* = 6). ^#^*p* < 0.05, compared with vehicle-treated control; ^∗^*p* < 0.05, significant compared with LPS alone; ^∗∗^*p* < 0.01, significant compared with LPS alone.

## References

[1] Butt Y., Kurdowska A., Allen T. C. (2016). Acute lung injury: a clinical and molecular review. *Archives of Pathology & Laboratory Medicine*.

[2] Papazian L., Calfee C. S., Chiumello D. (2016). Diagnostic workup for ARDS patients. *Intensive Care Medicine*.

[3] Liu S., Zhao Z., Tan L. (2020). Optimal mean airway pressure during high-frequency oscillatory ventilation in an experimental model of acute respiratory distress syndrome: EIT-based method. *Annals of Intensive Care*.

[4] Karagiannidis C., Joost T., Strassmann S. (2020). Safety and Efficacy of a novel Pneumatically Driven Extracorporeal Membrane Oxygenation device. *The Annals of Thoracic Surgery*.

[5] Morty R. E., Kuebler W. M. (2014). TRPV4: an exciting new target to promote alveolocapillary barrier function. *American Journal of Physiology. Lung Cellular and Molecular Physiology*.

[6] Seeley E. J., Rosenberg P., Matthay M. A. (2013). Calcium flux and endothelial dysfunction during acute lung injury: a STIMulating target for therapy. *The Journal of Clinical Investigation*.

[7] Tauseef M., Knezevic N., Chava K. R. (2012). TLR4 activation of TRPC6-dependent calcium signaling mediates endotoxin-induced lung vascular permeability and inflammation. *The Journal of Experimental Medicine*.

[8] Wang G., Zhang J., Xu C., Han X., Gao Y., Chen H. (2016). Inhibition of SOCs attenuates acute lung injury induced by severe acute pancreatitis in rats and PMVECs injury induced by lipopolysaccharide. *Inflammation*.

[9] Suresh K., Servinsky L., Reyes J. (2015). Hydrogen peroxide-induced calcium influx in lung microvascular endothelial cells involves TRPV4. *American Journal of Physiology. Lung Cellular and Molecular Physiology*.

[10] Kandasamy K., Bezavada L., Escue R. B., Parthasarathi K. (2013). Lipopolysaccharide induces endoplasmic store Ca2+-dependent inflammatory responses in lung microvessels. *PLoS One*.

[11] Lee C., Xu D. Z., Feketeova E. (2005). Store-operated calcium channel inhibition attenuates neutrophil function and postshock acute lung injury. *The Journal of Trauma*.

[12] Yin J., Michalick L., Tang C. (2016). Role of transient receptor potential vanilloid 4 in neutrophil activation and acute lung injury. *American Journal of Respiratory Cell and Molecular Biology*.

[13] Snutch T. P., Zamponi G. W. (2018). Recent advances in the development of T-type calcium channel blockers for pain intervention. *British Journal of Pharmacology*.

[14] Cribbs L. L. (2006). T-type Ca^2+^ channels in vascular smooth muscle: multiple functions. *Cell Calcium*.

[15] Bernhardt M. L., Zhang Y., Erxleben C. F. (2015). CaV3.2 T-type channels mediate Ca^2+^ entry during oocyte maturation and following fertilization. *Journal of Cell Science*.

[16] Todorovic S. M., Jevtovic-Todorovic V. (2011). T-type voltage-gated calcium channels as targets for the development of novel pain therapies. *British Journal of Pharmacology*.

[17] Mangoni M. E., Traboulsie A., Leoni A. L. (2006). Bradycardia and slowing of the atrioventricular conduction in mice lacking CaV3.1/alpha1G T-type calcium channels. *Circulation Research*.

[18] Rossier M. F. (2016). T-type calcium channel: a privileged gate for calcium entry and control of adrenal steroidogenesis. *Frontiers in Endocrinology (Lausanne)*.

[19] Zhou C., Wu S. (2006). T-type calcium channels in pulmonary vascular endothelium. *Microcirculation*.

[20] Zheng Z., Chen H., Xie P. (2019). *α*1GT-type calcium channel determines the angiogenic potential of pulmonary microvascular endothelial cells. *American Journal of Physiology-Cell Physiology*.

[21] Wu S., Haynes J., Taylor J. T. (2003). Cav3.1 (alpha1G) T-type Ca^2+^ channels mediate vaso-occlusion of sickled erythrocytes in lung microcirculation. *Circulation Research*.

[22] Zhou C., Chen H., Lu F. (2007). Cav3.1 (alpha1G) controls von Willebrand factor secretion in rat pulmonary microvascular endothelial cells. *American Journal of Physiology. Lung Cellular and Molecular Physiology*.

[23] Zhou C., Chen H., King J. A. (2010). *α*1GT-type calcium channel selectively regulates P-selectin surface expression in pulmonary capillary endothelium. *American Journal of Physiology. Lung Cellular and Molecular Physiology*.

[24] Chen H., Bai C., Wang X. (2014). The value of the lipopolysaccharide-induced acute lung injury model in respiratory medicine. *Expert Review of Respiratory Medicine*.

[25] Mulder P., Richard V., Compagnon P. (1997). Increased survival after long-term treatment with mibefradil, a selective T-channel calcium antagonist, in heart failure. *Journal of the American College of Cardiology*.

[26] Li-Mei W., Jie T., Shan-He W., Dong-Mei M., Peng-Jiu Y. (2016). Anti-inflammatory and anti-oxidative effects of dexpanthenol on lipopolysaccharide induced acute lung injury in mice. *Inflammation*.

[27] Bilici D., Nur Banoğlu Z., Kiziltunç A., Avci B., Çiftçioğlu A., Bilici S. (2002). Antioxidant effect of T-type calcium channel blockers in gastric injury. *Digestive Diseases and Sciences*.

[28] Qiu C., Bruneval P., Roeckel A., Heudes D., van Huyen J. P. D., Roux S. (1999). Mibefradil prevents L-NAME-exacerbated nephrosclerosis in spontaneously hypertensive rats. *Journal of Hypertension*.

[29] Egashira N., Okuno R., Abe M. (2008). Calcium-channel antagonists inhibit marble-burying behavior in mice. *Journal of Pharmacological Sciences*.

[30] Schingnitz U., Hartmann K., MacManus C. F. (2010). Signaling through the A2B adenosine receptor dampens endotoxin-induced acute lung injury. *Journal of Immunology*.

[31] Simonsen U., Wandall-Frostholm C., Olivan-Viguera A., Kohler R. (2017). Emerging roles of calcium-activated K channels and TRPV4 channels in lung oedema and pulmonary circulatory collapse. *Acta Physiologica*.

[32] Andres D., Keyser B., Benton B. (2016). Transient receptor potential (TRP) channels as a therapeutic target for intervention of respiratory effects and lethality from phosgene. *Toxicology Letters*.

[33] Zamponi G. W., Striessnig J., Koschak A., Dolphin A. C. (2015). The physiology, pathology, and pharmacology of voltage-gated calcium channels and their future therapeutic potential. *Pharmacological Reviews*.

[34] Parekh A. B., Putney J. W. (2005). Store-operated calcium channels. *Physiological Reviews*.

[35] Balakrishna S., Song W., Achanta S. (2014). TRPV4 inhibition counteracts edema and inflammation and improves pulmonary function and oxygen saturation in chemically induced acute lung injury. *American Journal of Physiology. Lung Cellular and Molecular Physiology*.

[36] Alvarez D. F., King J. A., Weber D., Addison E., Liedtke W., Townsley M. I. (2006). Transient receptor potential vanilloid 4-mediated disruption of the alveolar septal barrier: a novel mechanism of acute lung injury. *Circulation Research*.

[37] Weissmann N., Sydykov A., Kalwa H. (2012). Activation of TRPC6 channels is essential for lung ischaemia-reperfusion induced oedema in mice. *Nature Communications*.

[38] Gandhirajan R. K., Meng S., Chandramoorthy H. C. (2013). Blockade of NOX2 and STIM1 signaling limits lipopolysaccharide-induced vascular inflammation. *The Journal of Clinical Investigation*.

[39] Chong C. R., Sullivan D. J. (2007). New uses for old drugs. *Nature*.

[40] Santi C. M., Cayabyab F. S., Sutton K. G. (2002). Differential inhibition of T-type calcium channels by neuroleptics. *The Journal of Neuroscience*.

[41] Gomora J. C., Daud A. N., Weiergraber M., Perez-Reyes E. (2001). Block of cloned human T-type calcium channels by succinimide antiepileptic drugs. *Molecular Pharmacology*.

[42] Mohamed B. P., Goadsby P. J., Prabhakar P. (2012). Safety and efficacy of flunarizine in childhood migraine: 11 years’ experience, with emphasis on its effect in hemiplegic migraine. *Developmental Medicine and Child Neurology*.

[43] Tytgat J., Vereecke J., Carmeliet E. (1988). Differential effects of verapamil and flunarizine on cardiac L-type and T-type Ca channels. *Naunyn-Schmiedeberg's Archives of Pharmacology*.

[44] Tytgat J., Vereecke J., Carmeliet E. (1996). Mechanism of L- and T-type Ca2+ channel blockade by flunarizine in ventricular myocytes of the guinea-pig. *European Journal of Pharmacology*.

[45] Kuga T., Sadoshima J., Tomoike H., Kanaide H., Akaike N., Nakamura M. (1990). Actions of Ca2+ antagonists on two types of Ca2+ channels in rat aorta smooth muscle cells in primary culture. *Circulation Research*.

[46] Agoston A., Kunz L., Krieger A., Mayerhofer A. (2004). Two types of calcium channels in human ovarian endocrine cells: involvement in steroidogenesis. *The Journal of Clinical Endocrinology and Metabolism*.

[47] Wang C. S., Gao X. H., Cheng H. (2006). Effects of flunarizine on T-type calcium channels in mouse spermatogenic cells. *Zhonghua Nan Ke Xue*.

