# Supplementary material for: Mibefradil and Flunarizine, Two T-Type Calcium Channel Inhibitors, Protect Mice against Lipopolysaccharide-Induced Acute Lung Injury
Source: Mediators Inflamm. 2020 Nov 10;2020:3691701. doi: 10.1155/2020/3691701 (PMC7671802; doi:10.1155/2020/3691701)
Supplement: Supplementary Materials — Supplement figures: mibefradil (40 mg/kg) or saline was treated 30 min before aerosolized saline exposure, and mice were sacrificed 6 h after aerosol inhalation of saline. The total cell counts (a) in BALF, MPO activities (b) in lung tissue, total protein concentration (c) in BALF, extravasation of Evans blue dye (d) in lung tissue, TNF-α (e) and IL-6 (f) levels in BALF, pathological changes (g), and NF-κB activation in the lung were measured. All values are mean ± SEM (n = 6). #p < 0.05, compared with vehicle-treated control; ∗p < 0.05, significant compared with LPS alone; ∗∗p < 0.01, significant compared with LPS alone. [file 3691701.f1.doc]

Supplement figures:

A B

C D

E F

G

**M40**


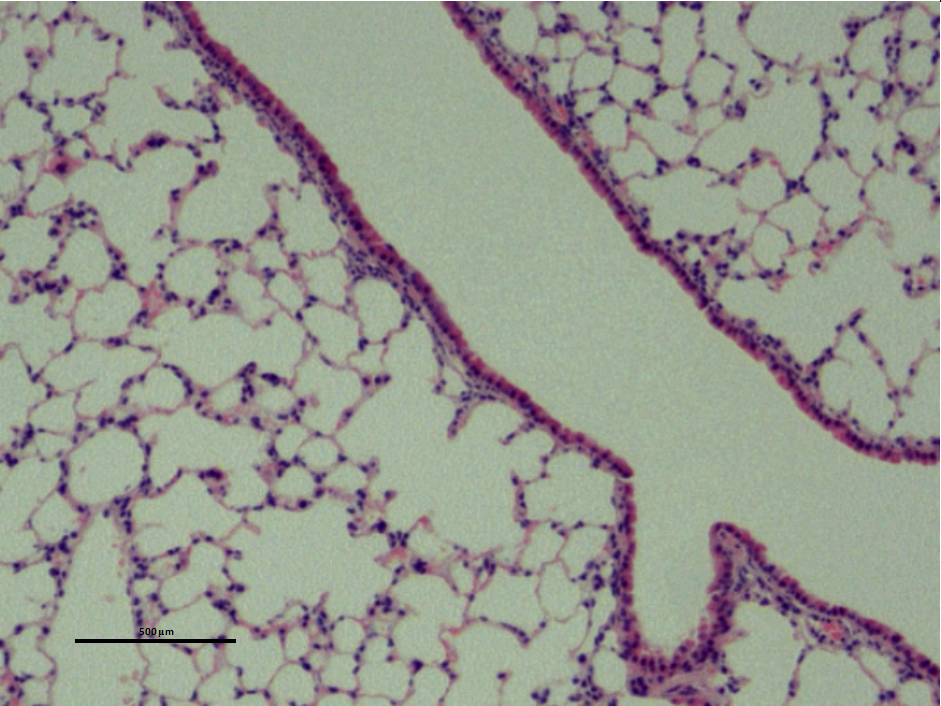


**Con**


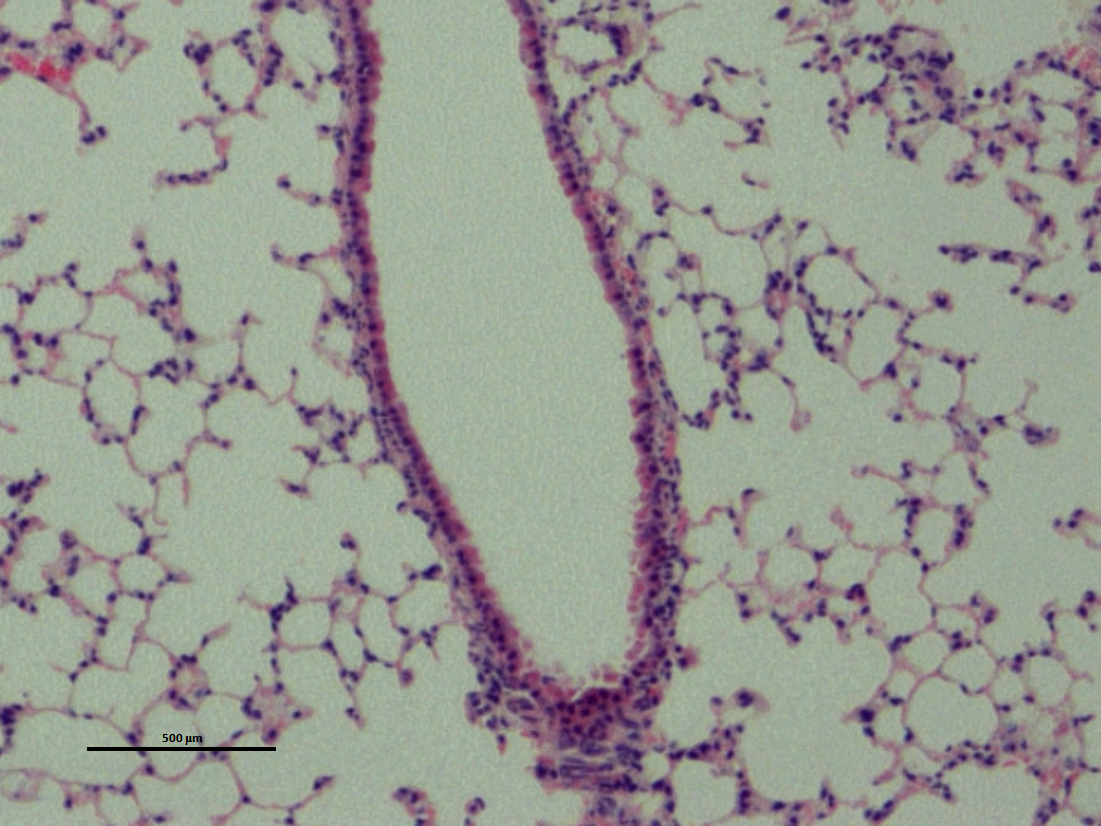


**M40**

I

Con

LPS+M40

LPS+M20

LPS

β-actin

IκB-α

p65

p-p65


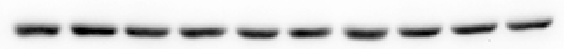

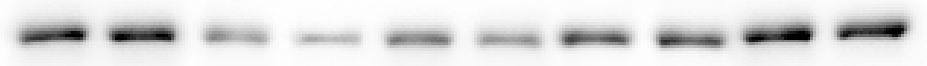

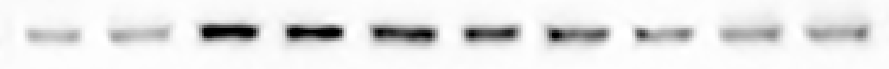

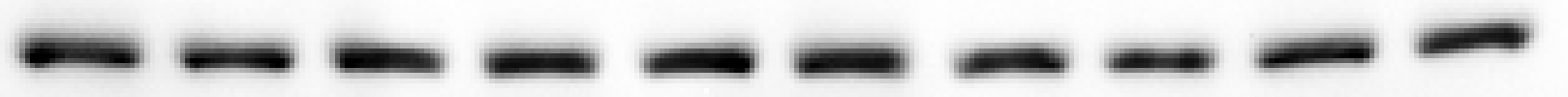


M40


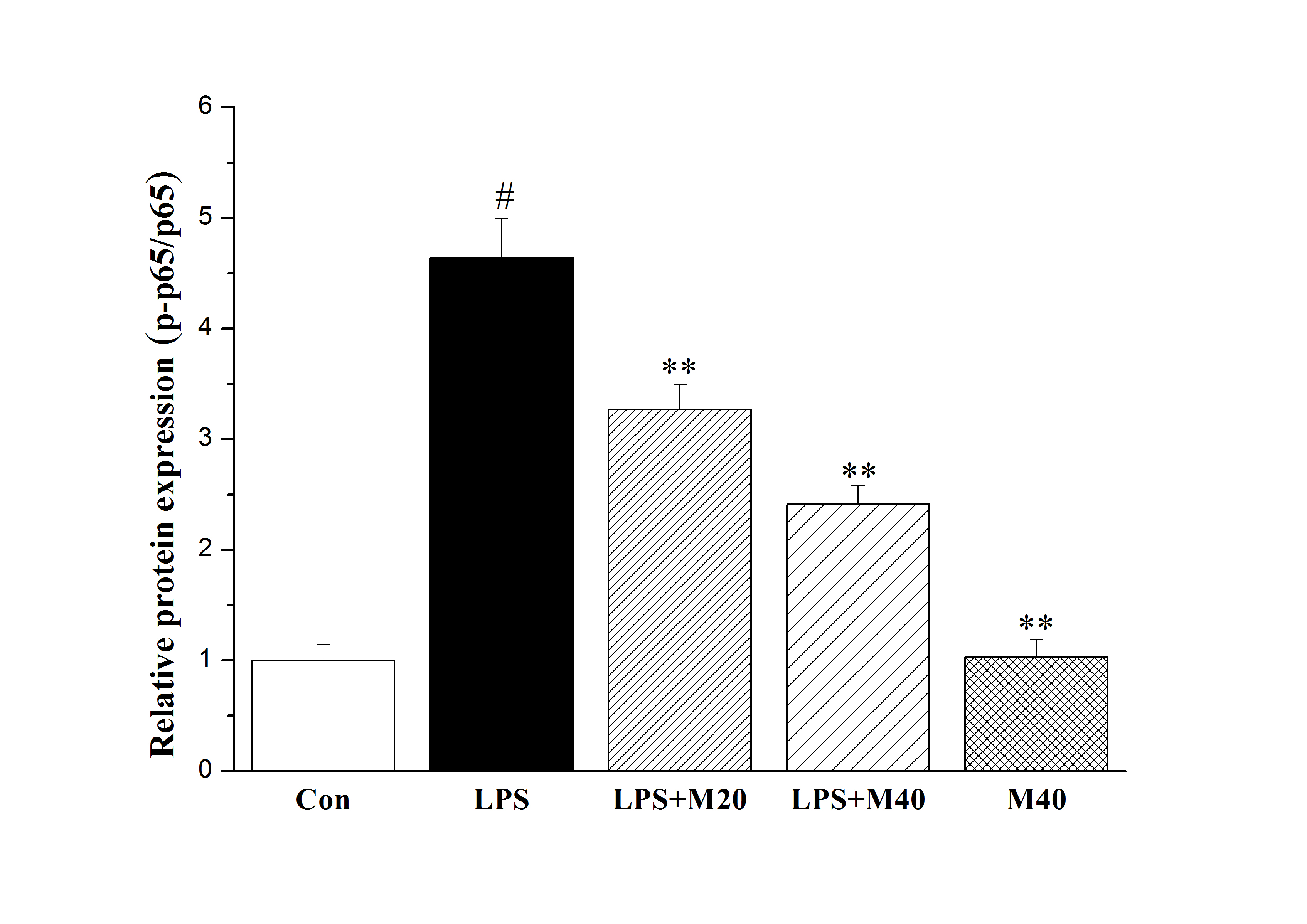

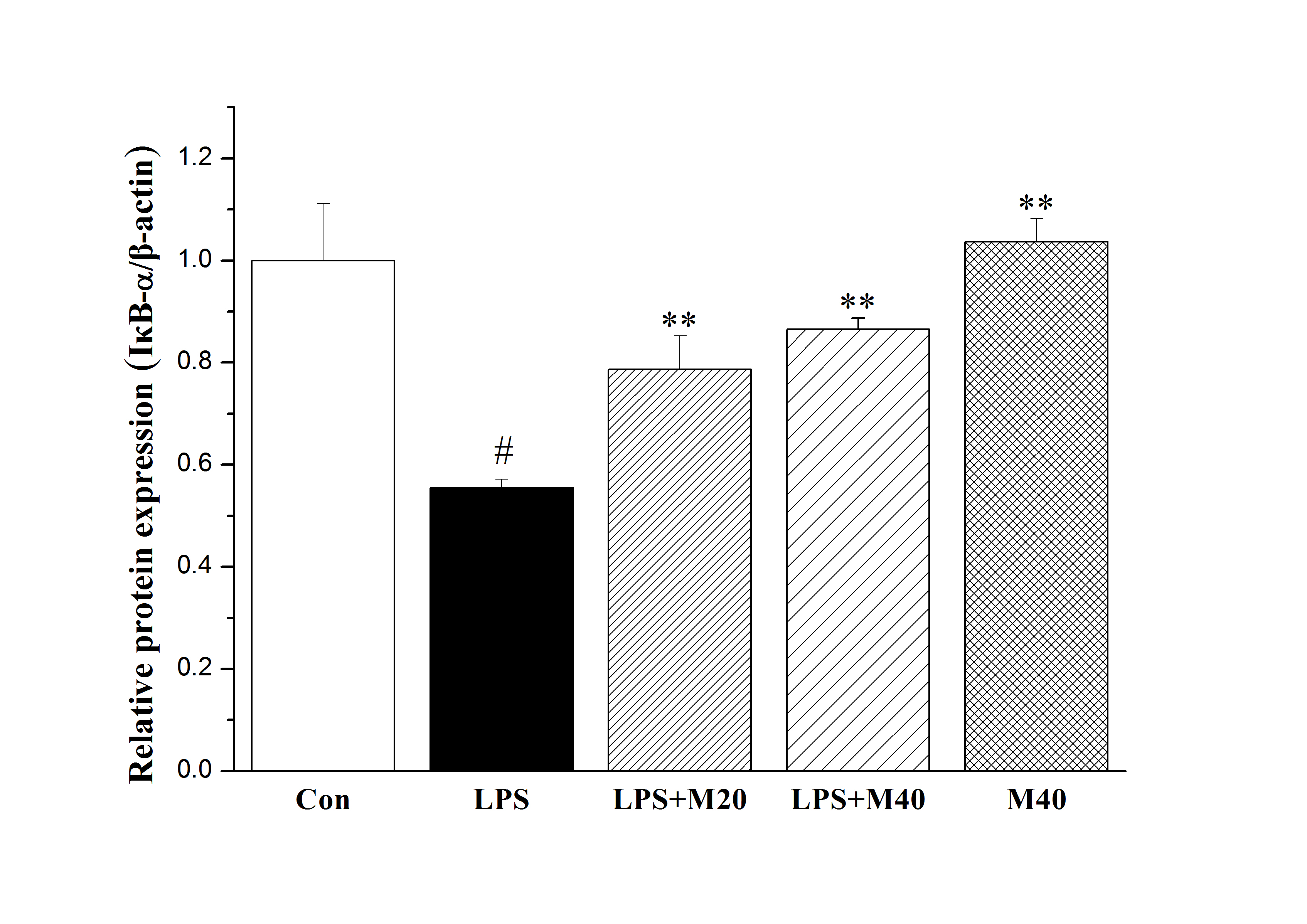


Supplement figures: Mibefradil (40 mg/kg) or saline was treated 30 min before aerosolized saline exposure, and mice were sacrificed 6 h after aerosol inhalation of saline. The total cell counts (A) in BALF, MPO activities (B) in lung tissue, total protein concentration (C) in BALF, extravasation of evans blue dye (D) in lung tissue, TNF-α (E) and IL-6 (F) levels in BALF, pathological changes (G) and NF-κB activation in the lung were measured. All values are mean ± SEM (n = 6). #p<0.05, compared with vehicle-treated control; *p<0.05, significant compared with LPS alone; **p<0.01, significant compared with LPS alone.
